# SELF-PERCEPTIONS OF AGING IN THE CONTEXT OF CHALLENGING EXPERIENCES: PATTERNS OF RISK AND RESILIENCE

**DOI:** 10.1093/geroni/igac059.375

**Published:** 2022-12-20

**Authors:** Hannah Giasson, Rachel Koffer, Jacqui Smith

## Abstract

Self-perceptions of aging have important implications for health and well-being in later life. Early life experiences, cultural and societal notions about age, and one’s present health and situational context may contribute to one’s expectations and perceptions of their own aging (e.g., Levy, 2009; Diehl et al., 2014; 2021). However, self-perceptions of aging may also predict people's responses in the face of current or future challenges. This symposium takes a closer look at self-perceptions of aging in the context of different types of life challenges. Hu and Larkina discuss early life informal caregiving experiences as antecedents of negative self-perceptions of aging in later life. Koffer and Giasson discuss how ten-year longitudinal associations between subjective age and future loneliness differ among current caregivers and non-caregivers. Mejia and colleagues discuss the role of self-perceptions of aging in adaptation to life following a fall, highlighting potential protective effects of positive self-perceptions of aging and sense of control. Finally, Giasson and colleagues discuss positive self-perceptions of aging as predictors of preventive health behavior and resilience in the context of the COVID-19 pandemic. Dr. Jacqui Smith will conclude the session with an integration of common themes, practical implications, and future research directions that emerge from the four studies.

